# Cerebrospinal Fluid Lactate as an Indicator for Post-neurosurgical Bacterial Meningitis

**DOI:** 10.5005/jp-journals-10071-23134

**Published:** 2019-03

**Authors:** Rebai Lotfi, Boussaidi Ines, Daghmouri M Aziz, Badri Mohamed

**Affiliations:** 1,2,3 Department of Anesthesiology and Critical Care Medicine, Burns and Trauma Center, Tunisia; 4 Department of Neurosurgery, Burns and Trauma Center, Tunisa

**Keywords:** Bacterial meningitis, CSF lactate, Neurosurgical

## Abstract

**Objective:**

To evaluate the interest of cerebrospinal fluid (CSF) lactate assay for the diagnosis of post-neurosurgical bacterial meningitis (PBM).

**Methods:**

We conducted at our neurosurgical resuscitation unit a prospective study of patients who underwent elective or emergency craniotomy. Lumbar puncture was performed in all patients who had clinical suspicion of PBM for CSF culture and cytological and chemical analysis (glucose, protein, lactate). The diagnosis of PBM is made according to the criteria proposed by the Center for Disease Control and Prevention (CDC). Receiver Operating Characteristic (ROC) was used to determine the diagnostic accuracy of CSF lactate.

**Results:**

72 patients were studied and only 32 of them had the clinical and biological criteria of the diagnosis of PBM. Median CSF lactate was 6.18 mmol/L for PBM vs 2.63 mmol/L for no PBM (p < 0.001). CSF lactate may predict the presence PBM, with a AUC of 0.98 and NPV of 99.1. The analysis of Youden's index also confirms the good diagnostic power of CSF lactate with a value of 83 at a cut-off value of 4 mmol/L and a sensitivity of 92.3% and specificity of 91.6%.

**Conclusion:**

Our study shows that the CSF lactate as an indicator for PBM. It is a fast and simple test that can help the clinician to optimize the management of PBM and decrease premature cessation of antibiotics.

**How to cite this article:**

Lotfi R, Ines B, et al. Cerebrospinal Fluid Lactate as an Indicator for Post-neurosurgical Bacterial Meningitis. Indian J Crit Care Med 2019;23(3):127-130.

## INTRODUCTION

Bacterial meningitis is relatively uncommon in post-neurosurgical^1^, but it is a serious and potentially life-threatening infection with a high mortality rate of 20 to 50%^2^. The diagnosis is difficult, the symptoms being frustrating and nonspecific in the postoperative period^3^. Contamination of the subarachnoid space with blood or degradation products resulting in a secondary inflammatory response, combined with antibiotic prophylaxis may make it difficult to interpret routine CSF analyses^4^. In addition, direct bacteriological examination results are often negative^5^. Several studies have shown that CSF lactate is a potential marker for identifying patients who develop bacterial meningitis^6^. The CSF lactate is a simple test and sensitive even in the case of blood contamination of the CSF^2^. The purpose of this study was to evaluate the efficacy of CSF lactate concentration in diagnosing PBM.

## METHODS

### Patients

We conducted a prospective study of patients admitted to Burns and Trauma Center, Ben Arous who underwent elective or emergency craniotomy between June 2015 and December 2016. Patients with external or internal CSF shunts were excluded. The patients were prospectively followed for the development of meningitis during the first 30 postoperative days. Lumbar puncture was performed with CSF sampling in all patients with clinical symptoms of bacterial meningitis such as fever, headache, and deterioration of neurological status within two to three days of craniotomy. Responsible parents of patients have been informed and received the signed consent from then.

Demographic data obtained included age, sex, Glasgow Coma Scale (GCS), indication for surgery, steroid use, antibiotic prophylaxis, operation duration, mechanical ventilation and length of UCI stay.

The diagnosis of PBM is made according to the criteria proposed by the Center for Disease Control and Prevention (CDC)^7^. Patients were divided into PBM group (Proven PBM and Presumed PBM) and no PBM group.

- Patient status was categorized following predefined criteria:
- **PBM:** positive bacterial CSF culture or Gram stainor Patient has at least 1 of the following symptoms with no other recognized cause: fever (>38 °C), headache, stiff neck, meningeal signs, cranial nerve signs, or irritability And positive CSF examination: CSF WBC count ≥100/ mm3 and C SF glucose <2.5 mmol/L or ratio of CSF glucose to blood glucose <0.4.
- **No PBM:** neither proven nor presumed criteria.

## MEASUREMENTS

CSF samples were obtained by lumbar puncture in a sterile manner. The CSF lactate and other biomarkers, including WBC count, CSF protein and CSF glucose were assessed quantitatively.

### Statistical Analysis

The statistical analyses were performed using SPSS software version 24. MedCalc statistical software was used to analyze the ROC curves.

The Kolmogorov-Smirnov test was used to determine the distribution of continuous variables. The medians of the CSF markers were compared using the non-parametric Mann-Whitney test. p value <0.05 was considered to be significant. The receiver operator curve (ROC) analysis was used to calculate the optimal cut-off value for CSF lactate. Youden index was used to define the diagnostic cut-off level for PBM.

## RESULTS

A total of 442 patients who underwent craniotomy in our center between June 2015 and December 2016 were identified. Among the 442 patients studied, 72 had a clinical suspicion of PBM and were divided into 32 patients to the PBM and 40 to the no PBM according to our preset criteria.

Demographic data are presented in Table 1. The incidence of PBM was 7.2%. The median age of those with and without meningitis were 47 years and 45 years, respectively. Brain tumor was the most common indication of surgery (44 patients). None of the following were associated with the development of meningitis: age, sex, operation duration, antibiotic prophylaxis, mechanical ventilation and length of UCI stay. The incidence of PBM was higher in steroid use group.

**Graph 1:**
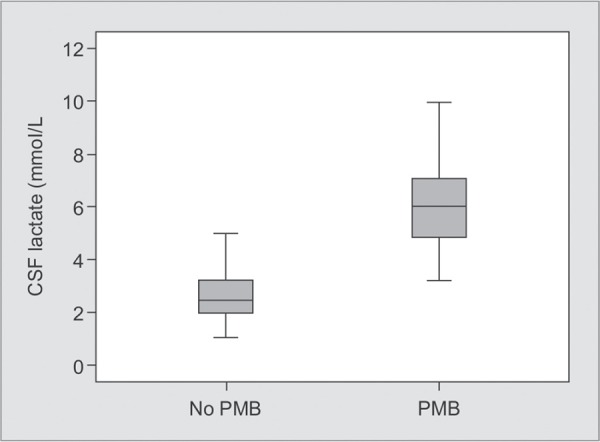
CSF lactate values for PBM and no PBM groups showing significant difference (Mann-Whitney, p < 0.001)

CSF markers data are presented in Table 2. In the PBM group, the median of the CSF lactate levels was 6.18 mmol/l, ranging from 3.1 mmol/L to 8.1 mmol/L, and was 2.63 mmol/L in the no PMB group, ranging from 1 mmol/L to 5.5 mmol/L (Graph 1).

The difference in the CSF lactate levels between the PBM and the no PBM groups was statistically significant (p < 0.001).

Median values of CSF leukocytes, proteins, glucose and CSF/ blood glucose ratio were also significantly higher in PBM than no PBM patients.

**Table 1:** Demographic data of patients

	*PBM*	*No PBM*
Age (yrs)	45 (29-62)	47 (26-71)
Sex		
Female	14 (43)	16 (40)
Male	18 (57)	24 (60)
GCS	13.1 (11-15)	13.5 (12-15)
Operation duration (h)	4.2 (3-6)	3.6 (3-5)
Antibiotic prophylaxis	32 (100)	37 (92)
Steroid use	20 (62)	18 (45)
Indication of surgery		
Tumor	19 (59)	25 (62.5)
Vascular disease	3 (9.3)	7 (17.5)
Trauma	10 (31.7)	8 (20)
Mechanical ventilation	3	2
Length of UCI stay (days)	4 (3-5)	3 (2-4)
Mortality	3 (9.3)	1 (2.5)

**Table 2:** CSF markers data

	*PBM*	*No PBM*	*p*
Glucose (mmol/l)	1.5 (0.4-2.6)	4.47 (3-6.2)	<0.001
CSF/blood glucose ratio	0.26 (0.1-0.39)	0.51 (0.41-0.66)	<0.001
Leucocytes (/mm3)	563(140-3000)	53 (20-100)	<0.001
Protein (g/l)	3.58 (0.9-6.6)	0.61 (0.2-1.2)	<0.001
Lactate (mmol/L)	6.18 (3.2-8.1)	2.63 (1-5.5)	<0.001

**Graph 2:**
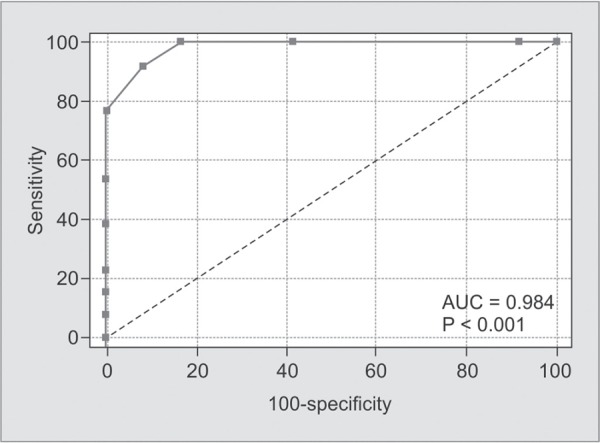
ROC curve analysis for CSF lactates

The ROC curve analysis of the CSF lactate data showed that the cut-off value was 4 mmol/L with AUC = 0.98, p < 0.001 (Graph 2). The analysis of Youden's index also confirms the good diagnostic power of CSF lactate, with a value of 83 at a cut-off value of 4 mmol/L, (sensitivity of 92.3, specificity of 91.6%, positive predictive value (PPV) 55.2 % and negative predictive value (NPV) 99.1%).

Five cultures of CSF samples were positive (21.8% of all meningitis). Gram-positive organisms predominated. Coagulase- negative Staphylococci were isolated from three patients, Pseudomonas aeruginosa from one patient and Klebsiella pneumonia from one patient.

## DISCUSSION

Our study confirms the value of CSF lactate assays for the diagnosis of PBM. An early and precise diagnosis of PBM is essential to start appropriate empiric antibiotic therapy. Since the CSF bacterial culture is routinely used as gold standard, the percentage of positive cultures does not exceed 10%, and this is due to several factors such as injection of antibiotics, contamination of the CSF sample^8–9^. The use of only bacterial culture may misidentify many patients with postoperative infections. Routine CSF analysis, such as glucose levels, cell counts and protein concentration are retained by most authors as criteria to diagnose PBM^10–12^. In many diagnostic studies in patients with PBM, CSF routine examinations, including WBC and neutrophil counts, protein levels and hypoglycorrhachia, were neither specific nor associated with high positive and negative predictive values^13^. Tamune et al. demonstrated that the CSF/blood glucose ratio may predict the presence of bacterial meningitis more precisely than other routinely measured markers in CSF^14^. The cell index (a ratio of WBC to RBC in the CSF divided by the ratio of WBC to RBC in blood) has been suggested to account for erythrocytes in the CSF because of hemorrhage. A cutoff value of 5 has been proposed to be indicative of infection but this has not been validated^15^.

Several studies have investigated the value of the CSF lactate assay to diagnose bacterial meningitis. Currently, it has been well established that astrocytes are able to produce lactate in response to a bacterial infection and that CSF lactate is little affected by serum lactate and therefore can be considered a good marker of intracranial infection^16^. In a recent meta-analysis, Xiao et al. have included 404 post-neurosurgical patients from 5 studies to evaluate the effectiveness of CSF lactate in the diagnosis of PBM^17^. It has shown that CSF lactate concentration has a good efficacy in the diagnosis of post-neurosurgical bacterial meneingitis with sensitivity at 0.92 and a specificity at 0.88^17^. In this meta-analysis, the cutoff values for CSF lactate ranged from 3.45 mmol/L to mmol/L (4.41 ± 0.85 mmol/L). Maskin et al.^11^. confirms, in 79 patients with signs of PBM that CSF lactate is relatively a good marker to distinguish between PBM and aseptic meningitis, even after antibiotic but the CSF lactate cutoff (4 mmol/L) value was defined before the analysis. Li et al^12^. retrospectively studied 178 patients and shows that the CSF lactate assay is very useful for the diagnosis of PBM. In their study, the cut-off value of CSF lactate was 3.45 mmol/L with higher sensitivity (90.0%) and specificity (84.6%), however, the author relied for the diagnosis of PBM on the old criteria cited in the study by Gray and Fedorko^18^. Our study shows the usefulness of CSF lactate for the diagnosis of PBM, with a AUC of 0.98 and NPV of 99.1. Youden Index for CSF lactate level was 0.83 at a cut-off value of 4 mmol/L, with a sensitivity of 92.3 and specificity of 91.6% confirming that CSF lactate concentration is a good indicator of PBM (Graph 1).

Previous recommendations proposed, if CSF Gram-stain and culture are negative, stopping of antimicrobial therapy; but this approach is not applicable with patients who have treated with antibiotics.

Our results show that if CSF lactate is lower 4 mmol/l, CSF glucose is normal and the Gram stain is negative, bacterial meningitis is unlikely. This algorithm can be used to decrease premature cessation of antibiotics.

Recently several studies have tested other markers such as procalcitonin, sCD163 and C-reactive protein but most of them were retrospective or with small sizes^19^.

In our study, coagulase-negative Staphylococci were the most commonly pathogens isolated in PBM group, which joins the literature data with a predominance of Gram-positive cocci pathogens in postneurosurgical infection^20^.

Our study presents some limitations. First it is a monocentric study with a small number of patients presenting a PBM. Second, the diagnosis of PBM according to the CDC criteria remains not applicable for patients with pre-existing neurologic deficits or who are sedated.

## CONCLUSION

Our study shows that the CSF lactate as an indicator for PBM. It is a fast and simple test that can help the clinician to optimize the management of PBM and decrease premature cessation of antibiotics. More investigation is needed to confirm our results.
